# Supplementary material for: Purchase and use of antimicrobials in the hospital sector of Vietnam, a lower middle-income country with an emerging pharmaceuticals market
Source: PLoS One. 2020 Oct 20;15(10):e0240830. doi: 10.1371/journal.pone.0240830 (PMC7575121; doi:10.1371/journal.pone.0240830)
Supplement: S1 Table — (DOCX) [file pone.0240830.s001.docx]

# S1 Table. List of available antimicrobials

| **J01AA_Tetracyclines** |
| --- |
| J01AA02_doxycycline |
| J01AA07_tetracycline |
| J01AA08_minocycline |
| J01AA12_tigecycline |
| **J01BA_Amphenicols** |
| J01BA01_chloramphenicol |
| **J01CA_Penicillins with extended spectrum** |
| J01CA01_ampicillin |
| J01CA04_amoxicillin |
| J01CA12_piperacillin |
| **J01CE_Beta lactamase sensitive penicillins** |
| J01CE01_benzylpenicillin |
| J01CE10_benzathine phenoxymethylpenicillin |
| **J01CF_Beta lactamase resistant penicillins** |
| J01CF02_cloxacillin |
| J01CF04_oxacillin |
| **J01CR_Combinations of penicillins, incl. beta lactamase inhibitors** |
| J01CR01_ampicillin and beta lactamase inhibitor |
| J01CR01p_ampicillin and beta lactamase inhibitor |
| J01CR02o_amoxicillin and beta lactamase inhibitor |
| J01CR02p_amoxicillin and beta lactamase inhibitor |
| J01CR03_ticarcillin and beta lactamase inhibitor |
| J01CR05_piperacillin and beta lactamase inhibitor |
| **J01DB_First generation cephalosporins** |
| J01DB01_cefalexin |
| J01DB03_cefalotin |
| J01DB04_cefazolin |
| J01DB05_cefadroxil |
| J01DB09_cefradine |
| J01DB12_ceftezole |
| **J01DC_Second generation cephalosporins** |
| J01DC01_cefoxitin |
| J01DC02_cefuroxime |
| J01DC03_cefamandole |
| J01DC04_cefaclor |
| J01DC07_cefotiam |
| J01DC09_cefmetazole |
| J01DC10_cefprozil |
| **J01DD_Third generation cephalosporins** |
| J01DD01_cefotaxime |
| J01DD02_ceftazidime |
| J01DD04_ceftriaxone |
| J01DD07_ceftizoxime |
| J01DD08_cefixime |
| J01DD10_cefetamet |
| J01DD12_cefoperazone |
| J01DD13_cefpodoxime |
| J01DD14_ceftibuten |
| J01DD15_cefdinir |
| J01DD62_cefoperazone and beta lactamase inhibitor |
| J01DD63_ceftriaxone and beta lactamase inhibitor |
| **J01DE_Fourth generation cephalosporins** |
| J01DE01_cefepime |
| J01DE02_cefpirome |
| **J01DH_Carbapenems** |
| J01DH02_meropenem |
| J01DH03_ertapenem |
| J01DH04_doripenem |
| J01DH51_imipenem and cilastatin |
| **J01EA_Trimethoprim and derivatives** |
| J01EA01_trimethoprim |
| **J01FA_Macrolides** |
| J01FA01_erythromycin |
| J01FA02_spiramycin |
| J01FA06_roxithromycin |
| J01FA09_clarithromycin |
| J01FA10o_azithromycin |
| J01FA10p_azithromycin |
| **J01FF_Lincosamides** |
| J01FF01_clindamycin |
| J01FF02_lincomycin |
| **J01GA_Streptomycins** |
| J01GA01_streptomycin |
| **J01GB_Other aminoglycosides** |
| J01GB01_tobramycin |
| J01GB03_gentamicin |
| J01GB04_kanamycin |
| J01GB06_amikacin |
| J01GB07_netilmicin |
| **J01MA_Fluoroquinolones** |
| J01MA01_ofloxacin |
| J01MA02_ciprofloxacin |
| J01MA03_pefloxacin |
| J01MA06_norfloxacin |
| J01MA07_lomefloxacin |
| J01MA12_levofloxacin |
| J01MA14_moxifloxacin |
| **J01MB_Other quinolones** |
| J01MB02_nalidixic acid |
| **J01R_COMBINATIONS OF ANTIBACTERIALS** |
| J01R_COMBINATIONS OF ANTIBACTERIALS |
| **J01RA_Combinations of antibacterials** |
| J01RA04_spiramycin and metronidazole |
| J01RA07_azithromycin, fluconazole and secnidazole |
| J01RA11_ciprofloxacin and tinidazole |
| J01RA13_norfloxacin and tinidazole |
| **J01XA_Glycopeptide antibacterials** |
| J01XA01_vancomycin |
| J01XA02_teicoplanin |
| **J01XB_Polymyxins** |
| J01XB01_colistin |
| **J01XD_Imidazole derivatives** |
| J01XD01_metronidazole |
| J01XD02_tinidazole |
| **J01XX_Other antibacterials** |
| J01XX01_fosfomycin |
| J01XX08_linezolid |
| J01XX09_daptomycin |
| **J02AA_Antibiotics** |
| J02AA01_amphotericin B |
| **J02AB_Imidazole derivatives** |
| J02AB02_ketoconazole |
| **J02AC_Triazole derivatives** |
| J02AC01_fluconazole |
| J02AC02_itraconazole |
| J02AC03_voriconazole |
| J02AC04_posaconazole |
| **J02AX_Other antimycotics for systemic use** |
| J02AX04_caspofungin |
| **J04AB_Antibiotics** |
| J04AB02_rifampicin |
| **J04AC_Hydrazides** |
| J04AC01_isoniazid |
| **J04AK_Other drugs for treatment of tuberculosis** |
| J04AK01_pyrazinamide |
| J04AK02_ethambutol |
| **J04AM_Combinations of drugs for treatment of tuberculosis** |
| J04AM02_rifampicin and isoniazid |
| J04AM05_rifampicin, pyrazinamide and isoniazid |
| J04AM06_rifampicin, pyrazinamide, ethambutol and isoniazid |
| **J05AB_Nucleosides and nucleotides excl. reverse transcriptase inhibitors** |
| J05AB01_aciclovir |
| J05AB06_ganciclovir |
| J05AB14_valganciclovir |
| **J05AF_Nucleoside and nucleotide reverse transcriptase inhibitors** |
| J05AF05_lamivudine |
| J05AF07_tenofovir disoproxil |
| J05AF08_adefovir dipivoxil |
| J05AF10_entecavir |
| **J05AG_Non nucleoside reverse transcriptase inhibitors** |
| J05AG03_efavirenz |
| **J05AH_Neuraminidase inhibitors** |
| J05AH02_oseltamivir |
| **J05AP_Antivirals for treatment of HCV infections** |
| J05AP01_ribavirin |
| J05AP03_boceprevir |
| **J05AR_Antivirals for treatment of HIV infections, combinations** |
| J05AR01_zidovudine and lamivudine |
| J05AR03_tenofovir disoproxil and emtricitabine |
| J05AR05_zidovudine, lamivudine and nevirapine |
| J05AR06_emtricitabine, tenofovir disoproxil and efavirenz |
| J05AR11_lamivudine, tenofovir disoproxil and efavirenz |
| **Other medication** |
| Other medication |
| **P01AB_Nitroimidazole derivatives** |
| P01AB01_metronidazole |
| P01AB02_tinidazole |
| P01AB07_secnidazole |
| **P01BA_Aminoquinolines** |
| P01BA01_chloroquine |
| **P02BA_Quinoline derivatives and related substances** |
| P02BA01_praziquantel |
| **P02BX_Other antitrematodal agents** |
| P02BX04_triclabendazole |
| **P02CA_Benzimidazole derivatives** |
| P02CA01_mebendazole |
| P02CA03_albendazole |
| **P02CF_Avermectines** |
| P02CF01_ivermectin |
