# Supplementary figures and images for: Purchase and use of antimicrobials in the hospital sector of Vietnam, a lower middle-income country with an emerging pharmaceuticals market
Source: PLoS One. 2020 Oct 20;15(10):e0240830. doi: 10.1371/journal.pone.0240830 (PMC7575121; doi:10.1371/journal.pone.0240830)

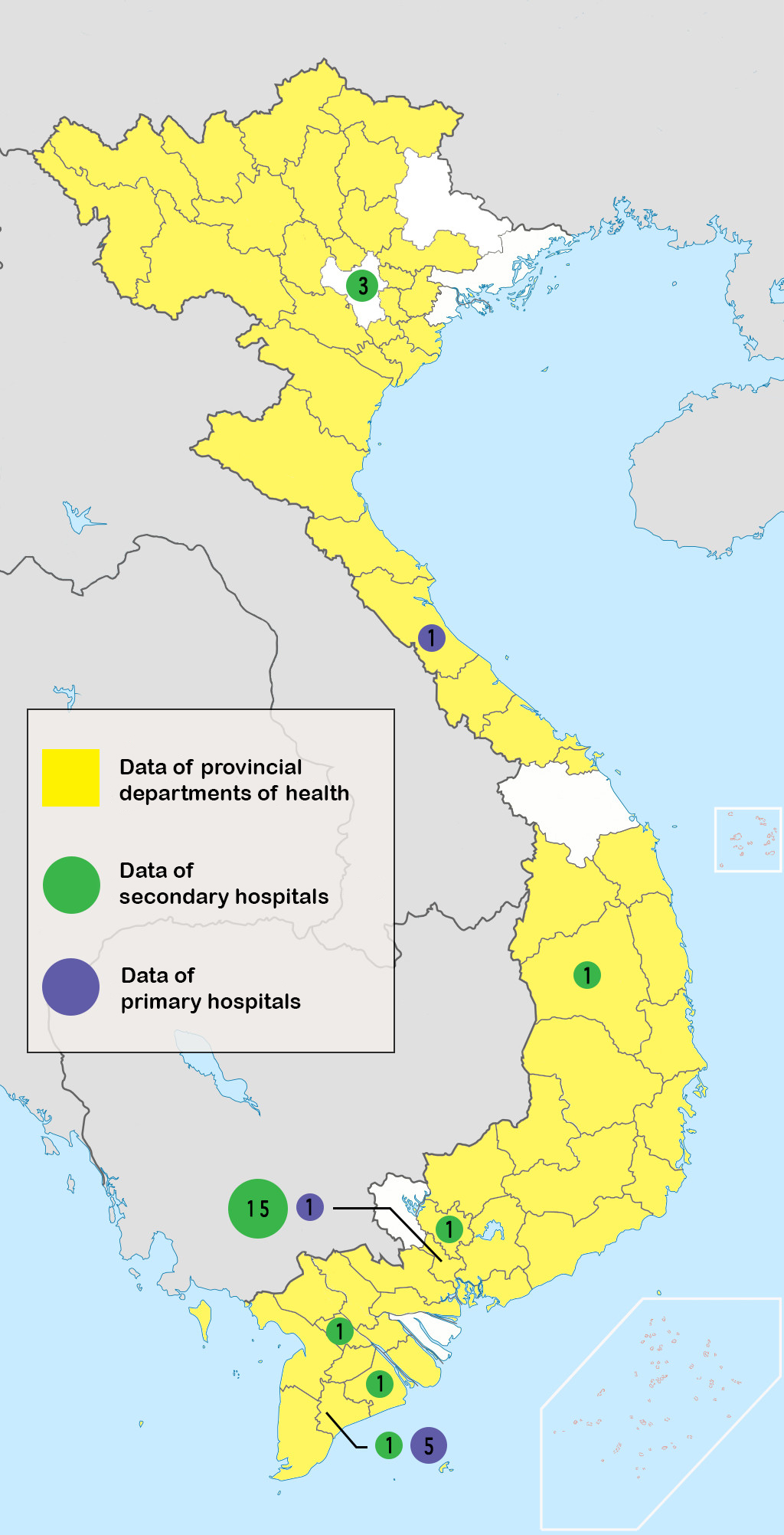

Supplement: S1 Fig — (JPG) [file pone.0240830.s002.jpg]
